# Technique for chest compressions in adult CPR

**DOI:** 10.1186/1749-7922-6-41

**Published:** 2011-12-10

**Authors:** Taufiek K Rajab, Charles N Pozner, Claudius Conrad, Lawrence H Cohn, Jan D Schmitto

**Affiliations:** 1 Department of Surgery, Brigham and Women's Hospital, Harvard Medical School, Boston, MA, USA; 2 Department of Emergency Medicine, Brigham and Women's Hospital, Harvard Medical School, Boston, MA, USA; 3 Department of Surgery, Massachusetts General Hospital, Harvard Medical School, Boston, MA, USA; 4 Division of Cardiac Surgery, Brigham and Women's Hospital, Harvard Medical School, Boston, MA, USA; 5 Department of Cardiac, Thoracic, Transplantation and Vascular Surgery, Hannover Medical School, Hannover, Germany

## Abstract

Chest compressions have saved the lives of countless patients in cardiac arrest as they generate a small but critical amount of blood flow to the heart and brain. This is achieved by direct cardiac massage as well as a thoracic pump mechanism. In order to optimize blood flow excellent chest compression technique is critical. Thus, the quality of the delivered chest compressions is a pivotal determinant of successful resuscitation. If a patient is found unresponsive without a definite pulse or normal breathing then the responder should assume that this patient is in cardiac arrest, activate the emergency response system and immediately start chest compressions. Contra-indications to starting chest compressions include a valid Do Not Attempt Resuscitation Order. Optimal technique for adult chest compressions includes positioning the patient supine, and pushing hard and fast over the center of the chest with the outstretched arms perpendicular to the patient's chest. The rate should be at least 100 compressions per minute and any interruptions should be minimized to achieve a minimum of 60 actually delivered compressions per minute. Aggressive rotation of compressors prevents decline of chest compression quality due to fatigue. Chest compressions are terminated following return of spontaneous circulation. Unconscious patients with normal breathing are placed in the recovery position. If there is no return of spontaneous circulation, then the decision to terminate chest compressions is based on the clinical judgment that the patient's cardiac arrest is unresponsive to treatment. Finally, it is important that family and patients' loved ones who witness chest compressions be treated with consideration and sensitivity.

## Introduction

Chest compressions have saved the lives of countless patients in cardiac arrest since they were first introduced in 1960 [1]. Cardiac arrest is treated with cardiopulmonary resuscitation (CPR) and chest compressions are a basic component of CPR. The quality of the delivered chest compressions is a pivotal determinant of successful resuscitation [2]. In spite of this, studies show that the quality of chest compressions, even if delivered by healthcare professionals, is often suboptimal [2]. Therefore it is important that providers carefully familiarize themselves with this technique.

## Indications

Chest compressions are generally indicated for all patients in cardiac arrest. Unlike other medical interventions, chest compressions can be initiated by any healthcare provider without a physician's order. This is based on implied patient consent for emergency treatment [3]. If a patient is found unresponsive without a definite pulse or normal breathing then the responder should assume that this patient is in cardiac arrest, activate the emergency response system and immediately start chest compressions [4]. The risk of serious injury from chest compressions to patients who are not in cardiac arrest is negligible [5], while any delay in starting chest compressions has grave implications for outcome. Due to the importance of starting chest compressions early, pulse and breathing checks were de-emphasized in the most recent CPR guidelines [4]. Thus, healthcare providers should take no longer than 10 seconds to check for a pulse. The carotid or femoral pulses are preferred locations for pulse checks since peripheral arteries can be unreliable.

## Contraindications

In certain circumstances it is inappropriate to initiate chest compressions. A valid Do Not Resuscitate (DNR) order that prohibits chest compressions is an absolute contra-indication. DNR orders are considered by the attending physician on the basis of patient autonomy and treatment futility.

The principle of patient autonomy dictates that competent patients have a right to refuse medical treatment [6]. Therefore a DNR order should be documented if patients do not wish to be treated with chest compressions. For patients with impaired decision-making, previous preferences should be taken into account when making decisions regarding DNR.

The principle of treatment futility dictates that healthcare providers are not obliged to provide treatment if this would be futile [6]. Therefore a DNR order should be documented if chest compressions would be unlikely to confer a survival benefit or acceptable quality of life. However, few criteria can reliably predict the futility of starting chest compressions.

If there is any uncertainty regarding DNR status then chest compressions should be started immediately while the uncertainties are addressed. Compressions may subsequently be terminated as soon as a valid DNR order is produced.

Of note, patients with implantable left ventricular assist devices [7-9] or patients with total artificial hearts or biventricular assist devices [10] who suffer cardiac arrest from device failure should be resuscitated using a backup pump (e.g. ECMO [11,12]) if this is available rather than with chest compressions.

## The Physiology of Chest Compressions

Chest compressions generate a small but critical amount of blood flow to the heart and brain. This significantly improves the chances of successful resuscitation [13]. However, the precise mechanism of blood flow during chest compressions has been controversial since the 1960s. The two main hypotheses are the external cardiac massage model and the thoracic pump model.

The external cardiac massage model suggests that chest compressions directly compress the heart between the depressed sternum and the thoracic spine [1]. This ejects blood into the systemic and pulmonary circulations while backward flow during decompression is limited by the cardiac valves. The external cardiac massage model is supported by radiographic evidence of direct compression of cardiac structures during chest compressions [14].

The thoracic pump model suggests that chest compressions intermittently increase global intra-thoracic pressure, with equivalent pressures exerted on vena cava, the heart and the aorta [9]. Thus blood is ejected retrograde from the intra-thoracic venous vasculature as well as antegrade from the intra-thoracic arterial vasculature and both arterial as well as venous pressures rise concomitantly. Therefore the presence of an arterial pulse in itself is not a reliable indicator of blood flow. This principle is illustrated by the fact that a ligated artery will continue to pulsate even in the absence of blood flow. However, the compliance of venous capacitance vessels is greater than the compliance of arterial resistance vessels. Therefore a pressure differential between the extra-thoracic arterial and venous sides of the vascular tree is formed. This pressure differential is but a fraction of the arterial pulse pressure, yet it is sufficient to drive some blood flow. The thoracic pump model is supported by arterial and venous pressure tracings demonstrating simultaneous peaks in venous and arterial pressures during chest compressions [15].

In toto, the available evidence suggests that both cardiac massage and the thoracic pump contribute to blood flow during chest compressions. Yet even excellent chest compressions can only generate a fraction of baseline blood flow [16]. Therefore the time during chest compressions contributes to the ongoing ischemic insult to the patient's heart and brain.

The brain is the organ most susceptible to decreased blood flow and suffers irreversible damage within 5 minutes of absent perfusion. The myocardium is the second most susceptible organ, with ROSC directly related to coronary perfusion pressures [17]. Therefore successful resuscitation with neurologically intact survival and ROSC critically depends on maintaining blood flow to the heart and brain via chest compressions.

## Technique for Chest Compression

Chest compressions consist of forceful and fast oscillations of the lower half of the sternum [1]. The technique of delivering chest compressions is highly standardized and based on international consensus that is updated in 5-year intervals [4,13,18].

### Patient Positioning

The patient in cardiac arrest should be placed in supine position with the rescuer standing beside the patient's bed or kneeling beside the patient's chest [18]. Adjustment of the bed height or standing on a stool allows leveraging the body weight above the waist for mechanical advantage. For optimal transfer of energy during chest compressions the patient should be positioned on a firm surface such as a backboard early in resuscitation efforts. This decreases wasting of compressive force by compression of the soft hospital bed. While re-positioning the patient, interruptions of chest compressions should be minimized and care should be taken to avoid dislodging any lines or tubes [13].

### Hand Position and Posture

Place the dominant hand over the center of the patient's chest [19]. This position corresponds to the lower half of the sternum. The heel of the hand is positioned in the midline and aligned with the long axis of the sternum. This focuses the compressive force on the sternum and decreases the chance of rib fractures. Next, place the non-dominant hand on top of the first hand so that both hands are overlapped and parallel. The fingers should be elevated off the patient's ribs to minimize compressive force over the ribs. Also avoid compressive force over the xiphisternum or the upper abdomen to minimize iatrogenic injury.

The previously taught method of first identifying anatomical landmarks and then positioning the hands two centimeters above the xiphoid-sternal notch was found to prolong interruptions of chest compressions without an increase in accuracy [20]. Similarly, the use of the internipple line as a landmark for hand placement was found to be unreliable [21]. Therefore these techniques are no longer part of the international consensus guidelines [4,13,18].

For maximum mechanical advantage keep your arms straight and elbows fully extended. Position your shoulders vertically above the patient's sternum. If the compressive force is not perpendicular to the patient's sternum then the patient will roll and part of the compressive force will be lost.

### Compression Rate and Interruptions

The blood flow generated by chest compressions is a function of the number of chest compressions delivered per minute and the effectiveness of each chest compression. The number of compressions delivered per minute is clearly related to survival [22]. This depends on the rate of compressions and the duration of any interruptions. Chest compressions should be delivered at a rate of at least 100 compressions per minute [4] since chest compression rates below 80/min are associated with decreased ROSC [2]. Any interruptions of chest compressions should be minimized. Legitimate reasons to interrupt chest compressions include the delivery of non-invasive rescue breaths, the need to assess rhythm or ROSC, and defibrillation [18]. Hold compressions when non-invasive rescue breaths are delivered [18]. Once an advanced airway is established there is no need to hold compressions for further breaths. High-quality compressions must also continue while defibrillation pads are applied and the defibrillator is prepared [13]. Aim to minimize interruption of chest compressions during the changeover of rescuers. Including all interruptions the patient should receive at least 60 compressions per minute [13].

### Compression Depth, Recoil and Duty Cycle

Compression depth should be at least 5 cm, since sternal depression of 5 cm and over results in a higher ROSC [18]. No upper limit for compression depth has been established in human studies but experts recommend that sternal depression should not exceed 6 cm [13].

After each compression, allow the chest to recoil completely. Incomplete recoil results in worse hemodynamics, including decreased cardiac perfusion, cerebral perfusion and cardiac output [23]. Complete recoil is achieved by releasing all pressure from the chest and not leaning on the chest during the relaxation phase of the chest compressions [13]. However, avoid lifting the hands off the patient's chest, since this was associated with a reduction in compression depth [24].

The duration of the compression phase as a proportion of the total cycle is termed duty cycle. Although duty cycles ranging between 20% and 50% can result in adequate cardiac and cerebral perfusion [25], a duty cycle of 50% is recommended because it is easy to achieve with practice [4]. Thus the duration of the compression phase should be equivalent to the duration of the decompression phase. If the patient has hemodynamic monitoring via an arterial line then compression rate, compression depth and recoil can be optimized for the individual patient on the basis of this data.

### Rotating Rescuers

The quality of chest compressions deteriorates over time due to fatigue [26]. Therefore the compressor should be rotated every two minutes [13]. Rotating compressors more frequently than this may have detrimental effects due to interruptions of chest compressions from the practicalities of the changeover [27]. Consider rotating compressors during any intervention associated with appropriate interruptions of chest compressions, for example when defibrillating. Every effort should be made to accomplish the switch in less than five seconds. For this purpose it may be helpful for the compressor performing chest compressions to count out loud [13]. If the rotating compressors can be positioned on either side of the patient, one compressor can be ready and waiting to relieve the working compressor in an instant [4].

## Termination of Efforts

Chest compressions are terminated following ROSC and unconscious patients with normal breathing are placed in the recovery position [28]. If there is no ROSC, then the decision to terminate efforts is based on the clinical judgment that the patient's arrest is unresponsive to treatment. This decision should be made by the physician leading the emergency response team after consultation with the members of the team. The factors that are considered include the time to initiate chest compressions, duration of chest compressions, initial arrest rhythm, age, comorbidities and any reversible causes of cardiac arrest such as drug overdose [3]. The duration of cardiac arrest is the most important prognostic factor [29]. In general, chest compressions should be continued at least as long as VF persists. Prolonged chest compressions are less likely to succeed if there is no ROSC within half an hour. However, case reports with exceptional ROSC are well documented and each decision to terminate efforts should be made individually. Any family members and patients' loved ones who witness chest compressions should be treated with consideration and sensitivity.

## Complications

Life-threatening complications of chest compressions are extremely rare [24]. Such complications occur less frequently than 1% [30-35]. If hypotension is noted following ROSC then cardiogenic shock and abdominal injury are the most important complications of chest compressions that should be considered [31]. Rib fractures are the most frequent complication, with an incidence of 1/3 at autopsy [30]. However, rib fractures were noted in only 2% of non-arrest patients who received chest compressions from a bystander [5]. Following successful ROSC all patients should be re-evaluated for resuscitation-related injuries [28].

## Summary

High quality chest compressions are proven to save lives. If an unresponsive patient has no definite pulse or is not breathing normally then the responder should assume that this patient is in cardiac arrest, activate the emergency response system and immediately start chest compressions. Push hard and fast over the center of the chest. Minimize interruptions of chest compressions and aggressively rotate compressors. Following successful ROSC place the patient in the recovery position and re-evaluate for resuscitation-related injuries. If there is no reasonable chance for ROSC then the decision to terminate efforts should be made by the leader of the emergency response team. Any family members witnessing chest compressions should be treated with sensitivity and respect.

## Competing interests

The authors declare that they have no competing interests.

## Authors' contributions

TKR conceived the study, performed literature search and drafted the manuscript. CNP participated in the design of the study, was involved in drafting the manuscript and is responsible revising it critically. CC was involved in drafting the manuscript and critically revised it. LHC participated in the design of the study, was involved in drafting the manuscript and is responsible revising it critically. JDS conceived of the study, was involved in drafting the manuscript and participated in its design and coordination. All authors read and approved the final manuscript.
